# Impact of sex in stroke thrombolysis: a coarsened exact matching study

**DOI:** 10.1186/s12883-015-0262-z

**Published:** 2015-02-10

**Authors:** Christian Hametner, Lars Kellert, Peter Arthur Ringleb

**Affiliations:** Department of Neurology, University of Heidelberg, Im Neuenheimer Feld 400, 69120 Heidelberg, Germany; Department of Neurology, Ludwig-Maximilians-University Munich, Großhadern, Marchioninistr. 15, 81377 Munich, Germany

**Keywords:** Acute ischemic stroke, Intravenous thrombolysis, Coarsened exact matching, Sex, Intracerebral hemorrhage

## Abstract

**Background:**

It is not established whether sex influences outcome and safety following intravenous thrombolysis (IVT) in acute stroke. As a significant imbalance exists between the baseline conditions of women and men, regression analysis alone may be subject to bias. Here we aimed to overcome this methodical shortcoming by balancing both groups using coarsened exact matching (CEM) before evaluating outcome.

**Methods:**

From our local prospective stroke database we analyzed consecutive patients who suffered anterior circulation stroke and received IVT from 1998 to 04/2013 (n = 1391, 668 female, 723 male). Data were preprocessed by CEM, balancing for age, NIHSS, lesion side, hypertension, diabetes, atrial fibrillation, smoking, coronary heart disease, and previous stroke, which yielded a matched cohort of 502 women and 436 men (n = 938). Outcome was estimated by adjusted binomial logistic regression analysis incorporating matched weights.

**Results:**

No effect of sex was seen to predict good outcome (OR 1.04, CI 0.76–1.43) or mortality (OR 1.13, CI 0.73–1.73). However, female sex was a strong independent predictor of symptomatic intracerebral hemorrhage (sICH – ECASS-II definition, OR 3.62, CI 1.77-7.41) and fatal ICH (OR 4.53, CI 1.61-12.7).

**Conclusion:**

In balanced groups, the two sexes showed comparable outcomes following IVT. A novel finding was the higher rate of sICH and fatal ICH in women. In this analysis we also demonstrate how CEM can reduce multivariate imbalance and thereby improve estimates, already in crude, but more importantly, in adjusted regression analysis. Further investigations of multicentre data with improved analytical approaches that yield balanced sex-groups are therefore warranted.

## Background

It is still not established whether sex has an impact on outcome in acute stroke patients who received intravenous thrombolysis (IVT). Former studies reported mainly equipoise in the 3-months outcome following IVT in women compared to men [1-3], but also a disadvantage for women was found [4]. Two studies found a greater incidence of bleeding complications in men [1,2]. However, all these studies have a critical bias in common. As sex is a nature-determined factor, (primary) randomization is obviously not possible. In addition, if covariates are very different between the sexes, the results of regression analysis alone can be misleading [5-7]. To overcome these issues in comparing the sexes, we improved the balance within the groups in a first step by coarsened exact matching (CEM) [8], thereby neglecting outcome and safety variables. To account for the remaining bias in covariates and to estimate outcome, we then performed adjusted regression analysis. This two-step approach is less prone to model misspecification and even more robust than are results based on the full unmatched data set [7,9,10].

### Aims

Improve multivariate balance between the sexes using coarsened exact matching (CEM) to investigate whether IVT treated women differ from IVT treated men with respect to outcome and safety.

## Methods

From our local prospective stroke database we analyzed clinical and imaging data of all consecutive patients who received IVT from 1998 to 04/2013 (n = 1501). Our prospective local stroke database was managed and this study implemented according to the STrengthening the Reporting of OBservational studies in Epidemiology (STROBE) statement for reporting case–control studies [11]. Data were collected as part of national and international quality-control programs. The retrospective analysis of the data lacks any treatment influence and therefore written informed consent and a formal ethical approval from the local ethics committee of the University of Heidelberg was waived. We excluded from further analysis 93 patients with posterior circulation stroke, 13 patients due to missing clinical follow-up, and 4 patients who died before follow-up imaging. Therefore, 1391 patients comprised the unmatched cohort. Three-month outcome was assessed either during an outpatient visit or a telephone interview using the mRS. Good outcomes were adjusted with respect to NIHSS score at presentation as previously described [12]: Presenting NIHSS scores of 1 to 7 a mRS score 0 at follow-up, presenting NIHSS scores of 8 to 14 mRS scores of 0 or 1, and presenting NIHSS scores above 14 mRS scores of 0–2 were counted as good outcome. Time to treatment was defined as time from symptom onset to start of IVT. Symptomatic intracerebral hemorrhage (sICH) was defined according to the definition of the ECASS-II trial [13]. Fatal ICH was defined as death caused most probably due to sICH following IVT. It has been shown recently that it is preferable to treat missing data by multiple imputation rather than listwise deletion in further processing (matching, multiple regression analysis) [14,15]. Therefore, Amelia II [16] for multiple imputation (m = 10) was used to further process all (n = 1391) instead of (only) 1126 patients. Covariates were imputed as follows: statin use (4.5%), antithrombotics (3.7%), oral anticoagulation (2.2%), thrombocytes (7.9%), systolic blood pressure (15%), and diastolic blood pressure (15.6%). Importantly, no nonlisted variables and no outcome variables were imputed. As recommended, each imputed data set was analyzed separately and combined at the end [16]. Groups of baseline characteristics were compared with the Student’s T-Test, the Mann–Whitney U-Test, or the Fisher’s Exact Test, as appropriate, and accounted for matched weights on matched group comparisons. In all statistical analyses, a p-value of 0.05 was considered significant. The following variables were then preprocessed using CEM: age, NIHSS, lesion side, hypertension, diabetes, atrial fibrillation, smoking, coronary heart disease, and previous stroke. The aim of matching is not to estimate, but rather to find better balance in the multidimensional distribution of covariates of the groups. This in turn reduces the degree of dependence on the estimation model of the outcome variable and therefore diminishes bias [10]. In detail, the CEM algorithm consists of three steps. First, desired variables of all patients are coarsened temporarily. Second, all patients of the initial cohort are sorted into strata on the basis of their coarsened variables. Third, only patients with strata containing at least one woman and one man are kept; others are discarded. Additionally, a weighting variable is generated to equalize the number of women and men in one stratum. CEM is a matching method of the class monotonic imbalance bounding [8]. This means that reducing imbalance in the empirical distribution in one covariate has no effect on any other covariates chosen for balancing, which represents a clear advantage of CEM over other matching methods [17]. Of course, only observed variables are accounted for in matching, and thus bias of omitted covariates cannot be eliminated. For balance checking Iacus and colleagues introduced the multivariate imbalance measure L1 [8]. Ranging from 0 to 1 - L1 is a relative magnitude depending on the data set and the selected covariates. The more the two distributions overlap, the more L1 decreases and trends to zero. The advantage of this two-step approach, first performing a matching solution and then an outcome estimation, is that it is more robust than, for example, regression analysis alone and also insensitive to selecting outcome model specifications arbitrarily, which is a common potential bias source [7,9,10]. In a final step, outcome was estimated by binomial logistic regression incorporating matched weights. Statistical analysis was performed using R [18-20] and SPSS (SPSS Inc., 21.0 for Windows).

## Results

**Figure 1:**
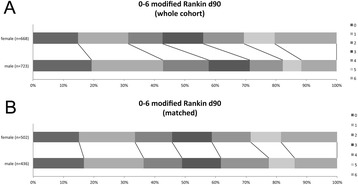
**mRS day 90 in (A) unmatched and (B) matched cohorts.**

The unmatched group comprised 668 woman and 723 men (n = 1391). Women were older than men (75.3y vs. 68.8y, p < 0.001) and suffered from more severe strokes according to NIHSS on admission (12 vs. 10, p < 0.001). Time to treatment (TTT) was equal for the two groups (140 min vs. 140 min, p = 0.615). In the female group hypertension (83.1% vs. 78.1%, p = 0.021) and atrial fibrillation (40.7 vs. 25.6%, p < 0.001) were observed more often, while current smoking (9.6% vs. 20.2%, p < 0.001), coronary heart disease (17.1% vs. 26.0%, p < 0.001) and hyperlipidemia (29.2% vs. 35.4%, p = 0.014) were less represented in women than in men. After improving balance, 502 women and 436 men (n = 938) comprised the matched cohort. Table 1 shows the baseline characteristics of the unmatched and matched cohort in detail. Unadjusted distribution of mRS for women and men prior to and after matching is presented in Figure 1. Multivariate imbalance measure L1 improved from 0.834 to 0.777.

**Table 1:** **Baseline characteristics for sex in unmatched and matched cohort (Matched variables are marked bold)**

	***Unmatched***			***Matched***		
	***Female***	***Male***	***P***	***Female***	***Male***	***P*** ^***§***^
N	668	723		502	436	
Age*	75.3 (12.1)	68.8 (12.7)	<0.001	74.4 (11.0)	74.3 (10.9)	0.939
**NIHSS** ^**†**^	12 (7; 17)	10 (6; 15)	<0.001	12 (7; 17)	12 (6; 17)	0.506
**Hemisphere, left** ^**‡**^	402 (60.2%)	412 (57.0%)	0.231	317 (63.1%)	275 (63.1%)	>0.999
**Hypertension** ^**‡**^	555 (83.1%)	565 (78.1%)	0.021	433 (86.3)	376 (86.3%)	>0.999
**Diabetes** ^**‡**^	170 (25.4%)	188 (26.0%)	0.854	106 (21.1%)	92 (21.1%)	>0.999
**Atrial fibrillation** ^**‡**^	272 (40.7%)	185 (25.6%)	<0.001	185 (36.9%)	161 (36.9%)	>0.999
**Current smoker** ^**‡**^	64 (9.6%)	146 (20.2%)	<0.001	33 (6.6%)	29 (6.6%)	>0.999
**CHD** ^**‡**^	114 (17.1%)	188 (26.0%)	<0.001	67 (13.3%)	58 (13.3%)	>0.999
**Previous Stroke** ^**‡**^	135 (20.2%)	155 (21.4%)	0.597	64 (12.7%)	56 (12.7%)	>0.999
TTT [min]^†^	140 (105; 180)	140 (105; 180)	0.615	140 (105; 180)	140 (110; 180)	0.504
Hyperlipidemia^‡^	195 (29.2%)	265 (35.4%)	0.014	142 (28.3%)	115 (26.3%)	0.557
Statin use^‡^	137 (20.5%)	202 (27.9%)	0.001	103 (20.5%)	96 (22.0%)	0.576
Antithrombotics^‡^	298 (44.6%)	340 (47%)	0.389	212 (42.2%)	216 (49.6%)	0.026
OAC^‡^	44 (6.6%)	31 (4.3%)	0.074	27 (5.4%)	21 (4.7%)	0.767
Systolic BP [mmHg]^†^	160.6 (22.1)	159.1 (22.1)	0.202	161.5 (21.0)	160.3 (22.5)	0.408
Diastolic BP [mmHg]^†^	86.1 (16.0)	88.2 (15.1)	0.009	86.1 (15.6)	86.3 (15.5)	0.856
Glucose [mg/dl]^†^	121 (105; 148)	120(105; 147)	0.434	120 (105; 147)	121 (106; 144)	0.706
Platelet [count/nl]^†^	263 (215; 313)	233 (193; 283)	<0.001	266 (216; 313)	234 (192; 277)	<0.001

Numbers are mean (standard deviation) or median (25/75 interquartile range) or counts (percentages).

NIHSS indicates National Institutes of Health Stroke Scale, CHD coronary heart disease, TTT time to treatment, OAC oral anticoagulation, BP Blood Pressure.

*Student’s T-Test.

^†^Mann Whitney-U Test.

^‡^Chi-Square/Fisher-Exact-Test.

^§^considering match weights.

### Good outcome

**Figure 2:**
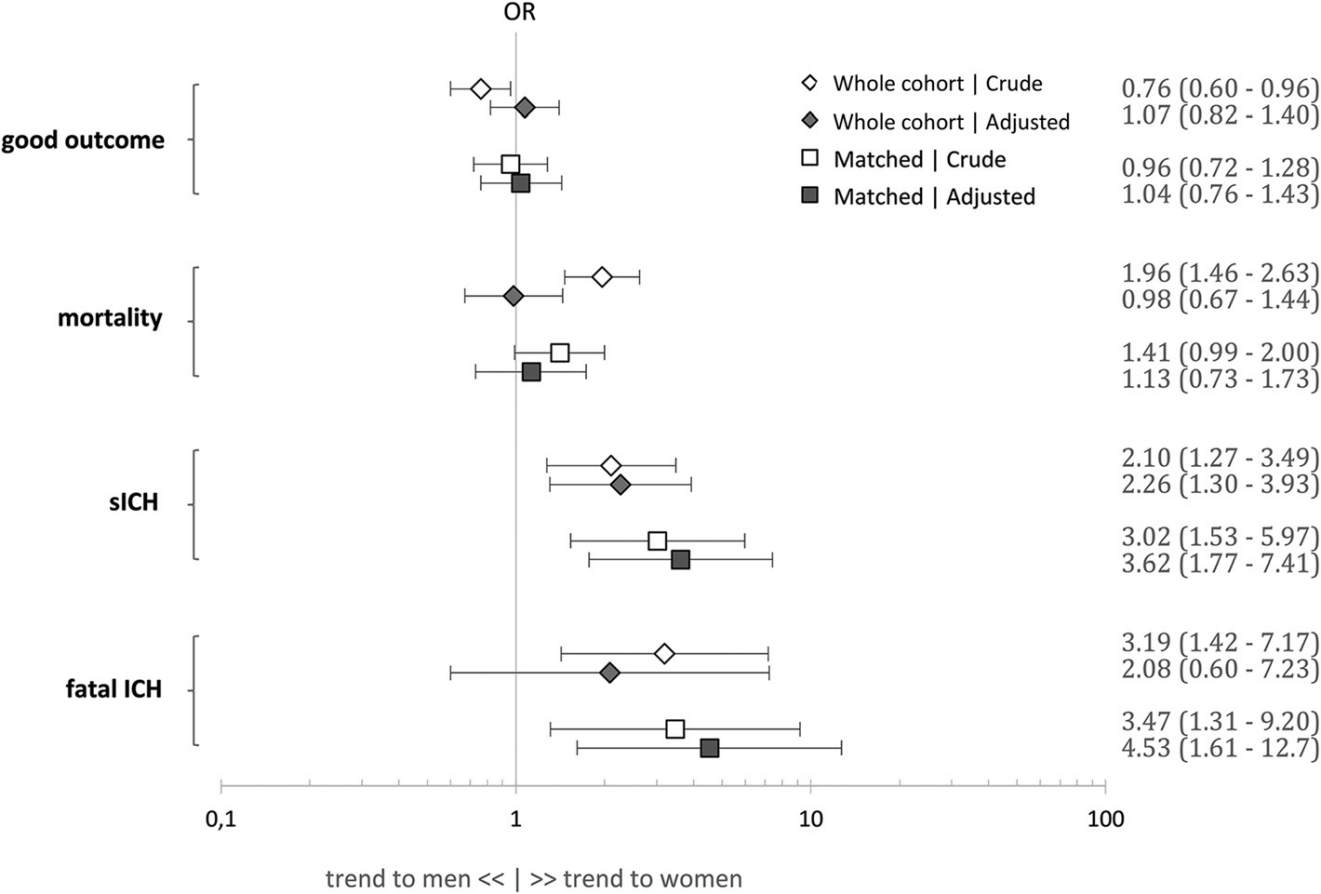
**Influence of sex on (1) good outcome, (2) mortality, (3) sICH, and (4) fatal ICH in unmatched (rhombus) and matched (square) cohorts performing crude (empty markers) and adjusted (filled markers) regression analysis.**

In the unmatched cohort, sex was significantly associated with good outcome (178 women (26.6%), 232 men (32.1%), p = 0.029) in the univariate, but not in the multivariate analysis (Figure 2). A logistic regression model to predict good outcome (139 women (27.7%), 124 men (28.3%), p = 0.827) in the matched cohort showed no effect of sex but did find an independent negative effect of age (OR 0.95, CI 0.93–0.97, p < 0.001), NIHSS (OR 0.94, CI 0.92–0.97, p < 0.001), TTT (OR 0.99, CI 0.99–0.99, p = 0.003), sICH (OR 0.14, CI 0.03–0.51, p = 0.002), current smoking (OR 0.49, CI 0.26–0.95, p = 0.035), and diastolic blood pressure (OR 0.98, CI 0.97–0.99, p = 0.007) (Table 2).

**Table 2:** Adjusted* binomial logistic regression full model in the matched cohort for (1) good outcome, (2) mortality, (3) sICH, and (4) fatal ICH

	**Good Outcome**		**Mortality**		**sICH**		**Fatal ICH**	
	**OR (95% CI)**	***P***	**OR (95% CI)**	***P***	**OR (95% CI)**	***P***	**OR (95% CI)**	***P***
Sex	1.04 (0.76–1.43)	0.774	1.13 (0.73–1.73)	0.572	3.62 (1.77–7.41)	<0.001	4.53 (1.61–12.7)	0.004
sICH	0.14 (0.03–0.51)	0.002	20.6 (9.52–44.7)	<0.001				
NIHSS	0.94 (0.92–0.97)	<0.001	1.10 (1.07–1.13)	<0.001	1.02 (0.98–1.07)	ns	1.03 (0.96–1.09)	ns
Age	0.95 (0.93–0.97)	<0.001	1.09 (1.06–1.12)	<0.001	0.97 (0.94–1.01)	ns	1.01 (0.95–1.06)	ns
TTT [min]	0.99 (0.99–0.99)	0.003	0.99 (0.99–0.99)	0.003	1.00 (1.00–1.00)	ns	1.00 (1.00–1.00)	ns
Diastolic BP [mmHg]	0.98 (0.97–0.99)	0.007	1.00 (0.99–1.02)	ns	0.99 (0.97–1.02)	ns	1.00 (0.97–1.03)	ns
Current Smoker	0.49 (0.26–0.95)	0.035	1.26 (0.37–4.20)	ns	0.07 (0.00–5.07)	ns	0.21 (0.00–15.2)	ns
Glucose [mg/dl]	0.99 (0.99–1.00)	ns	1.00 (1.00–1.01)	0.009	1.00 (0.99–1.01)	ns	1.00 (0.99–1.01)	ns
Hyperlipidemia	0.79 (0.49–1.27)	ns	1.99 (1.10–3.61)	0.022	1.21 (0.52–2.82)	ns	0.85 (0.24–3.07)	ns
Hypertension	1.21 (0.75–1.96)	ns	0.44 (0.21–0.90)	0.026	3.03 (0.64–14.3)	ns	0.49 (0.12–2.05)	ns
Antithrombotics	0.76 (0.52–1.11)	ns	1.29 (0.81–2.05)	ns	2.13 (1.04–4.37)	0.040	3.76 (1.26–11.2)	0.018
Atrial fibrillation	0.84 (0.58–1.21)	ns	1.10 (0.70–1.71)	ns	1.04 (0.53–2.01)	ns	1.48 (0.60–3.62)	ns
CHD	0.69 (0.39–1.21)	ns	1.73 (0.98–3.05)	0.055	0.91 (0.37–2.22)	ns	1.39 (0.46–4.16)	ns
Diabetes	0.99 (0.62–1.57)	ns	0.77 (0.44–1.37)	ns	2.01 (0.95–4.26)	0.070	1.32 (0.44–3.91)	ns
Previous Stroke	0.83 (0.49–1.39)	ns	1.35 (0.77–2.37)	ns	1.91 (0.89–4.10)	0.097	2.02 (0.73–5.62)	ns
OAC	1.58 (0.76–3.28)	ns	1.63 (0.68–3.90)	ns	0.11 (0.00–7.58)	ns	0.26 (0.00–20.2)	ns
Platelet [count/nl]	0.99 (0.99–1.00)	ns	1.00 (0.99–1.00)	ns	1.00 (0.99–1.00)	ns	1.00 (0.99–1.00)	ns
Hemisphere, left	0.83 (0.60–1.14)	ns	1.19 (0.76–1.84)	ns	0.62 (0.34–1.14)	ns	0.66 (0.29–1.52)	ns
Statin	0.95 (0.55–1.63)	ns	0.74 (0.37–1.49)	ns	0.61 (0.22–1.71)	ns	0.80 (0.15–4.10)	ns

*adjusted for NIHSS, lesion side, age, time to treatment (TTT), previous stroke, glucose, atrial fibrillation, hypertension, diabetes, hyperlipidemia, current smoker, coronary heart disease (CHD), antithrombotics, oral anticoagulation (OAC), platelet count, diastolic blood pressure (BP) and statin use, and symptomatic intracerebral hemorrhage (sICH, only in model 1 and 2).

### Mortality

Univariate analysis of mortality (137 women (20.5%), 84 men (11.6%), p < 0.001) in the unmatched group showed significant differences in sex, but the effect did not persist in multivariate analysis. Independently associated with mortality (93 women (18.5%), 61 men (13.9%), p = 0.064) in the matched cohort were age (OR 1.09, CI 1.06–1.12, p < 0.001), NIHSS (OR 1.10, CI 1.07–1.13, p < 0.001), TTT (OR 0.99, CI 0.99–0.99, p = 0.003), sICH (OR 20.6, CI 9.52–44.7, p < 0.001), glucose (OR 1.00, CI 1.00–1.01 p = 0.009), hypertension (OR 0.44, CI 0.21–0.90, p = 0.026), and hyperlipidemia (OR 1.99, CI 1.10–3.61, p = 0.022). Similarly to the unmatched cohort, a logistic regression model to predict mortality in the matched cohort showed no effect of sex (Table 2).

### sICH

Sex was significantly associated with sICH (45 women (6.7%), 24 men (3.3%), p = 0.004) in univariate and multivariate analysis in the unmatched cohort. Multivariate analysis to predict sICH (37 women (7.4%), 11 men (2.6%), p = 0.001) in the matched cohort found female sex (OR 3.62, CI 1.77–7.41 p < 0.001) and antithrombotic treatment (OR 2.13, CI 1.04–4.37, p = 0.04) as the only independent predictors (Table 2).

### Fatal ICH

Sex was significantly associated with fatal ICH (23 women (3.4%), 8 men (1.1%), p = 0.003) in univariate but not in the multivariate analysis in the unmatched cohort. Multivariate analysis to predict fatal ICH (20 women (4.0%), 5 men (1.2%) p = 0.008) in the matched cohort found female sex (OR 4.53, CI 1.61–12.7, p = 0.004) and antithrombotic treatment (OR 3.76, CI 1.26–11.2, p = 0.018) as the only independent predictors (Table 2).

## Discussion

To our knowledge this is the first time a balanced cohort of women and men has been used to analyse the influence of sex on outcome and safety after IVT in acute ischemic stroke. In these balanced groups 3-months outcome and mortality following IVT was comparable between the two sexes. In addition, we report the novel finding of increased bleeding complications in IVT-treated women.

We substantially tried to remove bias from our analysis of functional outcome. Saver and colleagues recently reported that it is meaningful to perform a baseline severity-adjusted endpoint analysis [12]. This adjustment may in particular be meaningful in a sex-based analysis, since NIHSS distributions may differ between the sexes, even if they appear similar in mean. The presented results appear to be in line with previous studies, which found no differences in functional outcome between the sexes evaluating mRS ≤ 1 [1,3] and mRS ≤ 2 [2]. However, they are not directly comparable, because of the adjustment chosen in our analysis. One single centre study also used a baseline severity adjustment evaluating mRS ≤ 2, but reported univariate results on sex only, because temperature was the main study focus [21].

Regarding mortality our results are in line with the post-hoc analysis of the Canadian Alteplase for Stroke Effectiveness Study [1], but contradict the previously largest study on this topic [2]. Lorenzano et al. found a higher mortality in women in univariate, but just the opposite, higher mortality in men, after multivariate adjustment. In our matched cohort, already univariate analysis yielded non-significant differences between the sexes (Figure 2), which were confirmed on additional adjustment. A limiting factor for a comparison of study-cohorts here might be the difference in analysis, namely different grades of multivariate balance and the differences in adjusting confounders. One example when estimating mortality is the consideration of sICH as a confounder. On the one hand sICH was not usually included in regression analysis for mortality of previous studies, although it is an established predictor for mortality [22,23]. Inclusion of sICH in the mortality regression may be misleading because mortality is also a part of the definition of sICH according to ECASS-II (any hemorrhage leading to death). However, on the other hand in our cohort female sex was an independent predictor of sICH. Therefore we preferred to include sICH in mortality regression analysis and thus omit an important sex-related mortality bias.

Again, crude mortality estimation of the matched cohort already gains more robust results that are not changed significantly after adjustment in contrast to estimation of the unmatched cohort. This demonstrates how researchers, if CEM is applied before regression analysis, may improve their estimates and how different study models may be better comparable, even if models slightly differ, because included confounders are chosen differently (see also [10]).

With respect to bleeding complication following IVT, female sex turned out to be the most important predictor for sICH and fatal ICH in our matched cohort. Previous studies observed a higher rate of sICH in men, reasoning that a higher incidence of antithrombotics and the higher absolute doses of recombinant tissue plasminogen activator (rtPA) (due to body weight) in men could account for this finding [2]. The intake of antithrombotics was lower in women in our cohort, thus favouring lower rates of sICH. Unfortunately, body weight was not consecutively registered, and therefore we can only conjecture very likely that the absolute dose of rtPA was higher in men. For the moment we are also interpreting the observed higher rate of sICH in women as being a single-centre phenomenon. However, external validation by a centre-based analysis of multicentre data including balanced sex cohorts should provide more in-depth insight regarding sex dependency on sICH.

The major strength of this study is its unique analytical approach, aiming to minimize the bias due to different covariates between the sexes. Pre-matching by CEM improves balance essentially and achieves more robust inferences than an unmatched, full data set does. An example of possible avoided bias is illustrated in Figure 2. In unmatched data, both crude and adjusted analyses either underestimate the effect (e.g., for sICH) or may give misleading results (e.g., for fatal ICH). However, facilitating a pre-matched regression analysis sex proved to be a strong independent predictor of fatal ICH in our cohort. Because CEM is a relatively novel rather than a standard approach we provide the reader with unmatched and matched outcome analysis to aim transparency and to enable a direct comparison of the results.

In our matching, we also included an often overlooked covariate: lesion side. By pathophysiological means, left and right anterior circulation strokes are reflected differently in the most commonly used score (NIHSS), with left-hemispheric strokes yielding higher scores but better outcomes [24]. Not considering these matters can produce a critical bias in determining outcome. This is the first study comparing sexes in stroke thrombolysis to address both the bias of side of lesion and baseline severity-adjusted analysis when determining outcome.

Our study has several limitations: This is a retrospective analysis of prospectively collected data from a single centre – external validation is needed. The matching process is accompanied by an attempt to find a reasonable compromise between the optimal match and the maximum size of the cohort. With respect to sICH we had no information regarding early infarct signs and we cannot adjust for body weight and consecutive rtPA dose. Our results are limited to patients eligible for treatment with IVT. Thus factors influencing outcome after stroke like older age and higher prestroke disability as well as sociodemographic parameters were not investigated in detail. We did not refer to parameters which are known to influence outcome after stroke like pre-stroke mRS, stroke subtype, vessel occlusion, and vessel recanalization. Outcome studies in stroke may be biased due to “do not resuscitate”- orders. This objection may therefore also be true for our cohort. In addition, there was no control group without IVT treatment. Therefore, we cannot conclude an absolute effect of IVT within sexes but only between the two sexes in comparison.

## Conclusion

In balanced groups, the two sexes show comparable outcomes following IVT. Taken together with the novel finding of higher rates of sICH and fatal ICH in women, further investigation of multicentre data in balanced groups is warranted. For observational data CEM seems to be a useful pre-processing tool to reduce bias in estimating outcome.
